# Aβ-induced degradation of BMAL1 and CBP leads to circadian rhythm disruption in Alzheimer’s disease

**DOI:** 10.1186/s13024-015-0007-x

**Published:** 2015-03-19

**Authors:** Hyundong Song, Minho Moon, Han Kyoung Choe, Dong-Hee Han, Changhwan Jang, Ahbin Kim, Sehyung Cho, Kyungjin Kim, Inhee Mook-Jung

**Affiliations:** Department of Biochemistry and Biomedical Sciences, College of Medicine, Seoul National University, 103 Daehak-ro, Seoul, 110-799 Jongno-gu Korea; Department of Biochemistry, College of Medicine, Konyang University, Daejeon, 302-718 Korea; Department of Biological Sciences, Seoul National University, Seoul, 151-742 Korea; Department of Neuroscience and Neurodegeneration Control Research Center, Kyung Hee University, Seoul, 130-701 Korea; Department of Brain Science, DGIST, Daegu, 711-873 Korea

**Keywords:** Alzheimer’s disease (AD), Amyloid-beta (Aβ), BMAL1 (Aryl hydrocarbon receptor nuclear translocator-like), CBP (Creb-binding protein), Circadian rhythm

## Abstract

**Background:**

Patients with Alzheimer’s disease (AD) frequently experience disruption of their circadian rhythms, but whether and how circadian clock molecules are perturbed by AD remains unknown. AD is an age-related neurological disorder and amyloid-β (Aβ) is one of major causative molecules in the pathogenesis of AD.

**Results:**

In this study, we investigated the role of Aβ in the regulation of clock molecules and circadian rhythm using an AD mouse model. These mice exhibited altered circadian behavior, and altered expression patterns of the circadian clock genes, *Bmal1* and *Per2*. Using cultured cells, we showed that Aβ induces post-translational degradation of the circadian clock regulator CBP, as well as the transcription factor BMAL1, which forms a complex with the master circadian transcription factor CLOCK. Aβ-induced degradation of BMAL1 and CBP correlated with the reduced binding of transcription factors to the *Per2* promoter, which in turn resulted in disruptions to PER2 protein expression and the oscillation of *Per2* mRNA levels.

**Conclusions:**

Our results elucidate the underlying mechanisms for disrupted circadian rhythm in AD.

**Electronic supplementary material:**

The online version of this article (doi:10.1186/s13024-015-0007-x) contains supplementary material, which is available to authorized users.

## Background

The circadian rhythm with a period of approximately 24 h is produced by an internal timekeeping mechanism referred to as the circadian clock machinery [1,2]. In mammals, the hypothalamic suprachiasmatic nucleus (SCN), a master circadian regulator, controls behavioral phenotypic rhythms and coordinates peripheral clocks located in a variety of organs such as the liver, kidney, and heart [3,4].

The physiological processes controlled by the circadian clock include sleep, hormone secretion, and glucose metabolism, which together determine daily circadian oscillation patterns. The circadian clock system also maintains multiple circadian oscillators in various organs to promote homeostatic balance and adaptability to the environment [5-7]. Disruption of the circadian rhythm in people, by shift work for example, significantly increases the risk of developing cardiovascular disease, cancer, neurodegenerative disorders, and metabolic syndrome, suggesting that circadian rhythm-controlled processes play critical roles in human physiology and in triggering disease processes [8,9].

Alzheimer’s disease (AD) is the most common age-dependent neurodegenerative disease. Aβ has been implicated as a key mediator of AD pathology, the levels of which are closely related with the neurodegeneration, memory deficits and neuronal cell loss [10]. Circadian rhythm disturbances are commonly reported in AD patients. More than 80% of AD patients over 65 years old suffer from circadian rhythm disorders, such as disturbances in thermoregulation and sleep-wake cycles [11,12]. Circadian disturbances become more severe as AD severity increases [12,13]. Since circadian disturbance is often noted early in the onset of AD in people, it may serve as a predictive tool or diagnostic indicator of developing AD pathology [13]; however, the underlying mechanisms linking Aβ with circadian rhythm disruption have not yet been elucidated.

In this study, we found that circadian behaviors such as home cage activity and body temperature were disrupted in 5XFAD mice modeling AD. The current observations have extended to previous studies using AD mouse models [14,15] and led us to hypothesize that Aβ is a major factor underlying circadian rhythm disruptions in 5XFAD mice. Furthermore, 5XFAD mice have consistently exhibited alterations to the oscillation patterns of circadian clock genes, such as the aryl hydrocarbon receptor nuclear translocator-like (*Bmal1*) and period circadian protein homolog 2 (*Per2*). These effects on circadian rhythm are correlated with the accelerated degradation of BMAL1 and Creb-binding protein (CBP) in 5XFAD mice as well as in HT22 cells. Importantly, we found that Aβ-induced BMAL1 degradation was mediated by the sumoylation of BMAL1, and that Aβ-induced CBP degradation was mediated by N-Cadherin cleavage products. Finally, the degradation of BMAL1 and CBP protein induced circadian rhythm disruption by dysregulating PER2 expression. These results demonstrate a critical role for Aβ in the disruption of the circadian clock in AD patients, and reveal a previously unknown link between Aβ and the degradation of BMAL1 and CBP.

## Results

### Abnormal circadian behavior in 5XFAD mice

**Figure 1:**
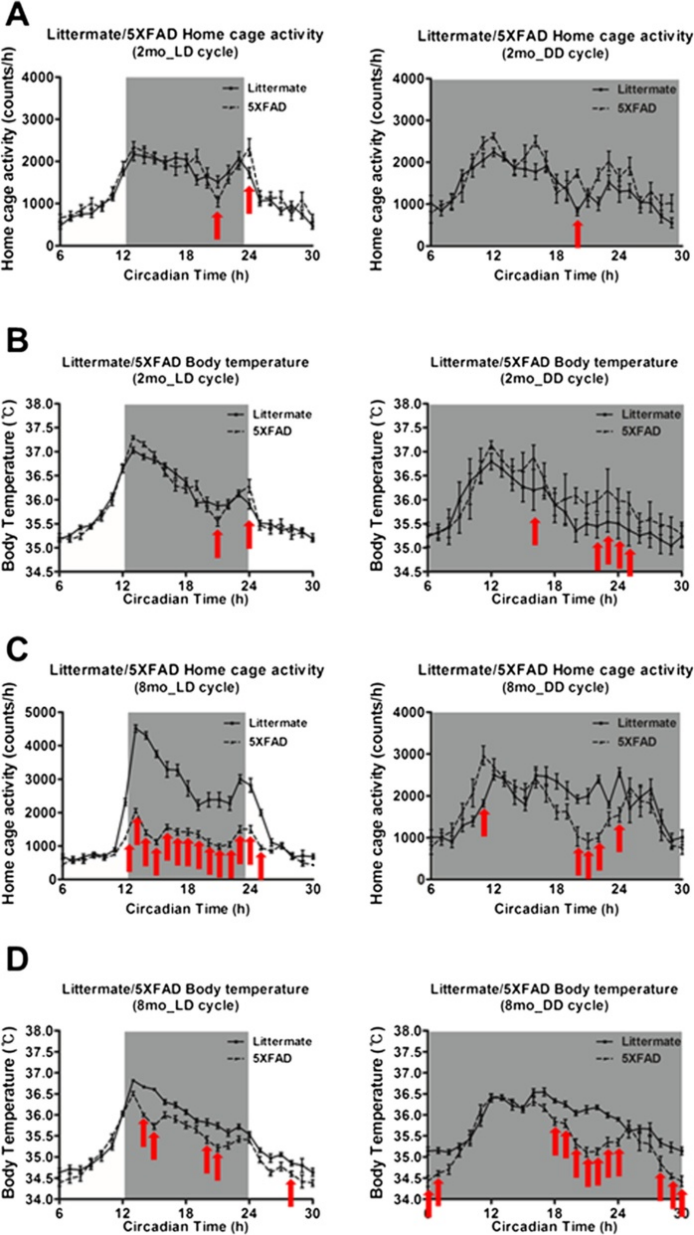
**Disrupted circadian rhythm in 5XFAD mice. (A & B)** Altered rhythmic waveform of home cage activity and body temperature in two-month-old 5XFAD mice in light–dark (LD) and dark-dark (DD) cycle. **(C & D)** Altered rhythmic waveform of home cage activity and body temperature in eight-month-old 5XFAD mice in LD and DD cycle.

Since previous studies have reported that circadian rhythms are disrupted in AD patients as well as in AD mouse models [14-17], we conducted behavioral assays to determine whether the circadian rhythms of 5XFAD mice are impaired. Both young (two months) and old (eight months) male mice, inserted with E-mitter probes, were entrained for one weeks to a 12 h:12 h Light–dark (LD) cycle with water and food available *ad libitum*. Their body temperature (BT) and home cage activity (HCA) were continuously measured at 6 min intervals, and their representative actograms are shown in Figure 1 and Additional file 1: Figure S1. Distinct daily patterns of BT and HCA were noticeable in young 5XFAD mice with change of BT and HCA at CT (Circadian Time) 19 and 24 h (Figure 1A,B; Additional file 2: Table S1; Additional file 3: Table S2; Additional file 1: Figure S1A,B). To test whether old 5XFAD mice exhibit additive behavioral disruption of increased circadian time, we conducted circadian behavioral assays. Old 5XFAD mice showed dramatically disrupted daily patterns in circadian behavior of both BT (CT 14, 15, 20, 21 and 29 h) and HCA (CT 12 to 25 h) compared with old littermate mice (Figure 1C,D; Additional file 2: Table S1; Additional file 3: Table S2; Additional file 1: Figure S1A,B). Since the abnormal circadian behavior in 5XFAD mice did not affect light-induced behavior, we conducted behavioral assays in constant darkness (DD) cycle. Both young and old 5XFAD mice also exhibited an altered BT and HCA in DD cycle compared with their littermates (Figure 1A-D; Additional file 2: Table S1; Additional file 3: Table S2; Additional file 1: Figure S1A–D), and two-way ANOVA revealed that these altered daily rhythms of BT and HCA are the results of interactions between the genotype (difference between 5XFAD mice and littermate mice) and circadian time (Table 1). These results suggest that Aβ may affect circadian rhythm in these mice.

**Table 1:** **ANOVA F and p values for BT and HCA rhythms**

**Physiological indices**		**Factors**		
		**Circadian time**	**Genotype**	**Interaction**
Young LD	Home cage activity	F(24,576)=49.97; ****p<0.0001	F(1,576)=0.58; ^n.s.^p=0.4526	F(24.576)=2.34; ****p<0.000l
	Body temperature	F(24,576)=14356; ****p<0.000l	F(1,576)=0.02; ^n.s.^p=0.8802	F(24,576)=311; ****p<0.0001
Young DD	Home cage activity	F(23,276)=l3.77; ****p<0.0001	F(1,275)=32.60: ****p<0.0001	F(23,276)=l.57; ^n.s.^p=0051
	Body temperature	F(24,288)=47.07; ****p<0.0001	F(1,288).67.53; ****p<0.0001	F(24,288)=3.42; ****p<0.0001
Old LD	Home cage activity	F(24.528)=101.23; ****p<0.000	F(1.528)=1818.65; ****p<0.000l	F(24.528)=30.03; ****p<0.0001
	Body temperature	F(24.528=143.35; ****p<0.0001	F(1,528)=40.62; ****p<0.0001	F(24.528)4.34; ****p<0.0001
Old DD	Home cage activity	F(24.288)=14.88; ****p<0.0001	F(1.288)=13.82; **p=0.00029	F(24.288)=4.56; ****p<0.0001
	Body temperature	F(24.288)=57.73; ****p<0.0001	F(l,288)=133.24; ****p<0.0001	F(24.288)=6.02; ****p<0.0001

Significant differences (**p,0.01, ****p<0.0001, n.s.=not significant) are indicated in bold type.

ANOVA, analysis of variance.

### The expression of molecular circadian clock genes, *Bmal1*, *Cbp* and *Per2*, are impaired in 5XFAD mice

**Figure 2:**
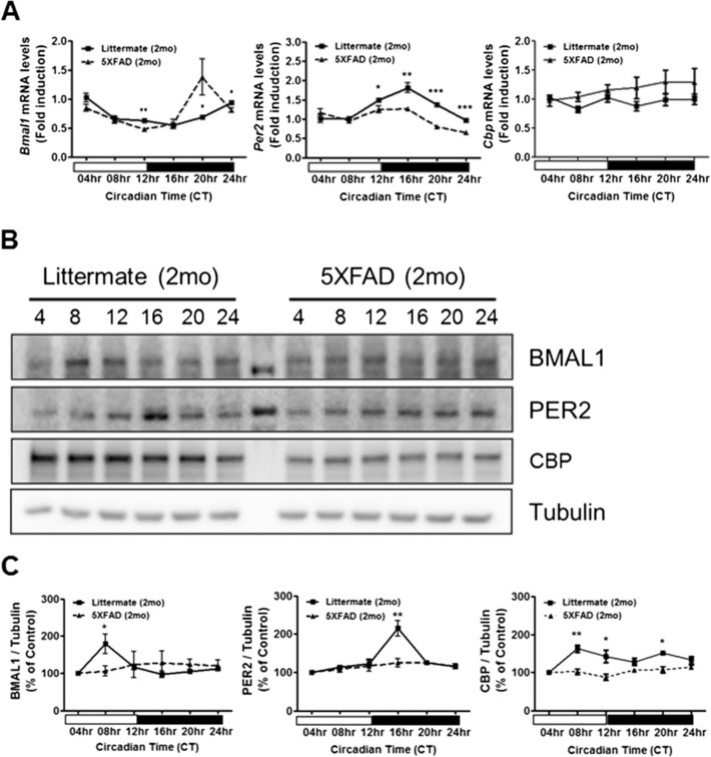
**Altered clock molecule expression in the SCN of 5XFAD mice. (A)** Temporal changes in mRNA levels of clock genes in the SCN of 5XFAD mice. The total RNA of the SCN was prepared at 4 h intervals. The mRNA levels of *Bmal1, Cbp* and *Per2* were quantified using real-time PCR. Data are represented as mean ± SEM; ^*^ *P* < 0.05, ^**^ *P* < 0.01, ^***^ *P* < 0.001. **(B)** Temporal changes in the protein levels of clock proteins in the SCN of 5XFAD mice. Brain samples were prepared at 4 h intervals. Western blot analysis revealed that BMAL1, CBP and PER2 protein oscillations were decreased in 5XFAD mice. **(C)** Densitometric analysis of BMAL1, CBP and PER2 protein expression from three independent experiments. BMAL1, CBP and PER2 were normalized to α-Tubulin. Data are represented as mean ± SEM. ^*^ *P* < 0.05, ^**^ *P* < 0.01.

To investigate the connection between the circadian behavioral disturbance and circadian clock genes, expression of molecular circadian clock genes such as *Bmal1, Cbp* and *Per2* was examined in the SCN of 5XFAD mice (2-month-old). We first performed qRT-PCR analysis from mice hypothalamic samples containing the SCN. We found that the levels of *Bmal1* and *Per2* mRNA in the SCN of 5XFAD mice were significantly altered, and showed abnormal circadian oscillations compared with those of control littermates (Figure 2A). However, *Cbp* mRNA levels were not altered between littermates and 5XFAD mice (Figure 2A). We therefore analyzed oscillations in the protein levels of endogenous circadian clock molecules in control and in 5XFAD mice (2-month-old). In contrast to their littermates, 5XFAD mice showed no noticeable oscillation patterns in the levels of BMAL1, CBP and PER2 proteins (Figure 2B,C). These results indicate that the 5XFAD mice have impairments in the regulation of circadian clock genes.

### Aβ induces the degradation of BMAL1 and CBP

**Figure 3:**
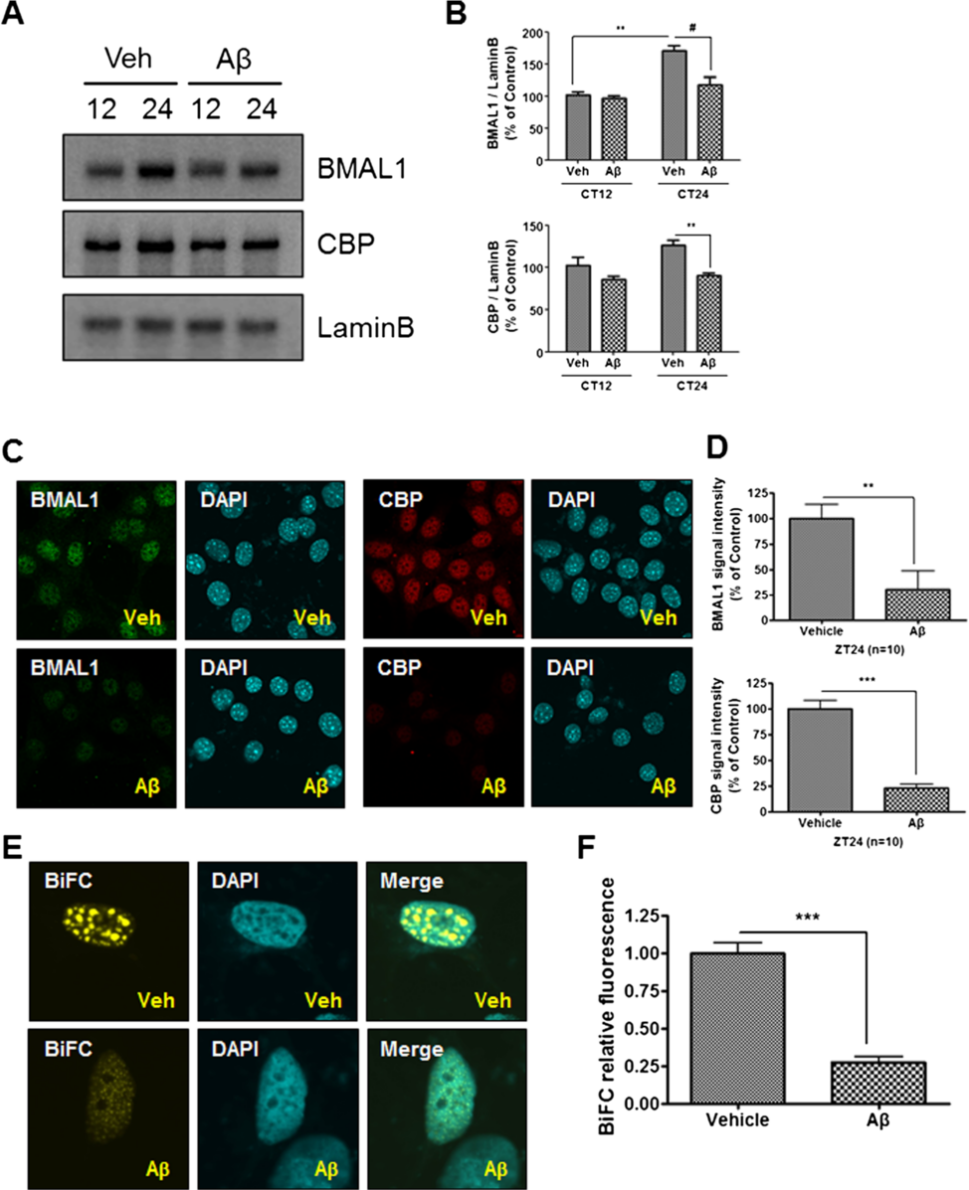
**Aβ induced BMAL1 and CBP protein degradation in the nucleus of HT22 cells** ***.*** **(A-D)** Aβ-induced BMAL1 and CBP protein degradation was measured in the nucleus of HT22 cells. **(A)** HT22 cells were incubated with vehicle or Aβ (2 μM) for CT12, 24 after cell synchronization. Western blot analysis showed that BMAL1 and CBP protein expression was decreased in the nucleus at CT24. **(B)** Densitometric analysis of BMAL1 and CBP protein expression from three independent experiments. The intensities of the bands from BMAL1 and CBP proteins were normalized to Lamin B. Data are represented as mean ± SEM. ^**^ *P* < 0.01, ^#^ *P* < 0.05. **(C)** Photomicrograph of immunostaining with BMAL1 and CBP proteins in HT22 cells clearly shows that BMAL1 and CBP immunofluorescence are significantly decreased within the nucleus following treatment with 2 μM of Aβ for 24 h. **(D)** Densitometric analysis of BMAL1 and CBP immunofluorescence from three independent experiments. Data are represented as mean ± SEM. ^**^ *P* < 0.01, ^***^ *P* < 0.001. **(E)** Aβ inhibited the formation of BMAL1-CBP BiFC complexes. Cos7 cells were transiently transfected with VC-BMAL1 and VN-CBP. 12 h post-transfection with VN-CBP and VC-BMAL1, cells were serum starved for 12 h and incubated with vehicle or Aβ for 24 h after cell synchronization. **(F)** Densitometric analysis of BMAL1-CBP BiFC complexes from three independent experiments. Data are represented as mean ± SEM. ^***^ *P* < 0.001.

It has been shown that CBP, a well-known circadian clock control molecule [18], is associated with the circadian transcriptional co-activator, BMAL1 [19]. To determine the functional relationship between CBP and BMAL1 in cultured cells, cellular clocks were synchronized in HT22 cells using a pulse treatment with Dexamethasone (Dex), which synchronize the cellular clock [20], followed by a 12, 24 h treatment with 2 μM Aβ. We found that the levels of BMAL1 and CBP proteins in Aβ-treated cells were significantly lower compared with those in vehicle-treated cells at CT24 as determined using western blot (Figure 3A,B) and immunofluorescence assays (Figure 3C,D). To investigate the dynamic changes in the molecular interactions between BMAL1 and CBP in living cells under physiological conditions, we employed the BiFC assay using an enhanced yellow fluorescence protein, Venus, which allows the visualization of protein interactions in living cells [21]. Cos7 cells were transiently transfected with VC (C-terminal)-BMAL1 and VN (N-terminal)-CBP. At 12 h post-transfection with VN–CBP and VC–BMAL1, respectively, the two groups of cells were serum-starved for 12 h, followed by synchronization and incubated with vehicle or Aβ for 24 h. We found that Aβ-induced degradation of BMAL1 and CBP correlates with disruption of the interactions between VC-BMAL1 and VN-CBP (Figure 3E,F). These results were also confirmed using BMAL1-CBP immunoprecipitation (Additional file 4: Figure S2). Real-time PCR analysis of the two groups showed no significant difference in the transcription of *Bmal1* and *Cbp* mRNAs in HT22 cells (Additional file 5: Figure S3A,B). These results collectively suggest that Aβ impacts on the metabolic stabilities of BMAL1 and CBP proteins.

### The degradation of BMAL1 by Aβ involves the sumoylation of BMAL1

**Figure 4:**
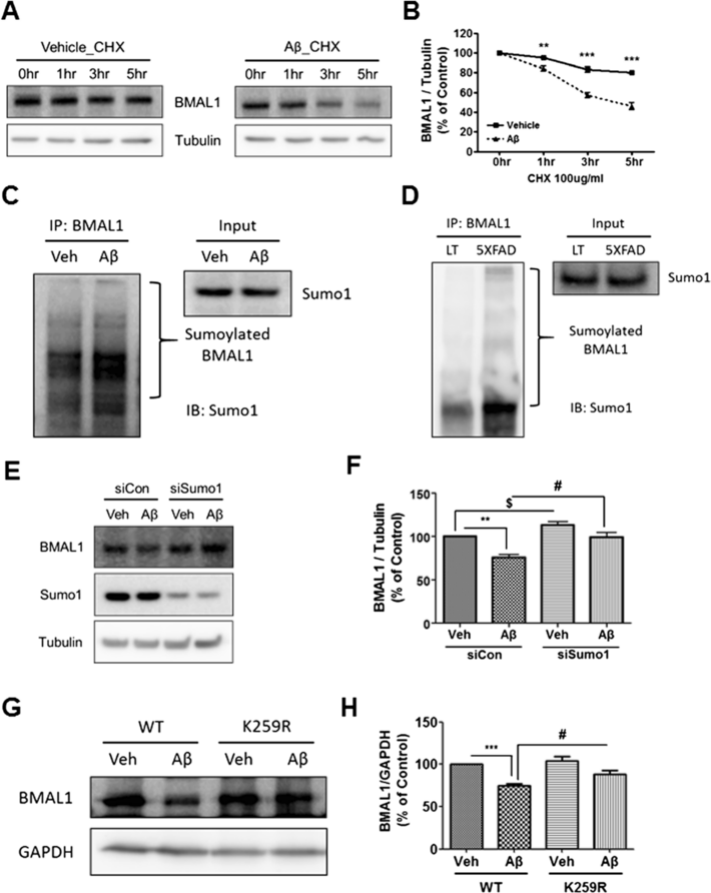
**Regulation of BMAL1 protein stability by Aβ-mediated sumoylation. (A & B)** Aβ affected the stability of BMAL1 protein. HT22 cells were incubated with CHX (100 μg/ml) with vehicle, or CHX (100 μg/ml) with Aβ (2 μM) for the indicated times. The protein level of BMAL1 was analyzed by immunoblotting with anti-BMAL1 antibody and then quantified by densitometry. Data are represented as mean ± SEM. ^**^ *P* < 0.01, ^***^ *P* < 0.001. **(C & D)** Effects of Aβ on the interaction between BMAL1 and SUMO1. **(C)** HT22 cells were incubated with vehicle or Aβ (2 μM) for 24 h after cell synchronization. **(D)** Both littermate and 5XFAD mice brains were prepared for immunoprecipitation. Each sample was immunoprecipitated using the anti-BMAL1 antibody. Confirmation by western blot analysis with anti-SUMO-1 antibody showed that the immunoprecipitated BMAL1 was physically interacted with SUMO1. This interaction was greater with Aβ treatment. **(E)** The transfection of *Sumo1* siRNA into HT22 cells rescued Aβ-induced BMAL1 degradation compared to control siRNA. **(F)** Densitometric analysis of BMAL1 protein from three independent experiments. BMAL1 was normalized to α-Tubulin. ^**^ *P <* 0.01, ^#^ *P <* 0.05, ^$^ *P <* 0.05. **(G)** The transfection of BMAL1 K259R into HT22 cells decreased Aβ-induced BMAL1 degradation compared to BMAL1 WT. **(H)** Densitometric analysis of BMAL1 protein from three independent experiments. BMAL1 was normalized to GAPDH. ^***^ *P <* 0.001, ^#^ *P <* 0.05.

Sumoylation is a post-translational modification implicated in diverse cellular systems, such as apoptosis, protein stability, transcriptional regulation, and nuclear-cytosolic transport [22]. It has been reported that sumoylation controls the molecular clock and the stability of BMAL1 [23]. To determine whether the stability of BMAL1 is regulated by Aβ, we monitored the degradation rate of BMAL1 in HT22 cells using the cycloheximide (CHX) chase assay. HT22 cells expressing GFP-BMAL1 were treated with CHX, followed by time-course immunoblotting (at 1, 3, and 5 h). GFP-BMAL1 was more rapidly degraded in Aβ-treated cells compared with vehicle-treated cells (Figure 4A,B). We further examined whether sumoylation of BMAL1 is influenced by Aβ treatment. Immunoprecipitation assays revealed that Aβ further induced sumoylation of BMAL1 compared with vehicle-treated cells (Figure 4C). To examine the role of Aβ in BMAL1 sumoylation in vivo, we used hippocampus in 5XFAD mice. Immunoprecipitation with BMAL1 specific antibody followed by immunoblotting with SUMO1 specific antibody showed that 5XFAD mice increased the sumoylation of BMAL1 compared with littermate mice (Figure 4D). We hypothesized that the absence of SUMO1 might reduce the Aβ-induced degradation of BMAL1 in HT22 cells. We silenced *Sumo1* function using *Sumo1* siRNA and measured the level of BMAL1 in Aβ-treated cells. In *Sumo1* siRNA-transfected HT22 cells, BMAL1 degradation was significantly diminished compared with control siRNA-transfected HT22 cells (Figure 4E,F). It is reported that BMAL1 is sumoylated on a lysine 259 (Lys^259^) residue [23,24]. To determine whether Aβ-induced degradation of BMAL1 could be affected by sumoylation, we transiently expressed wild-type BMAL1 (BMAL1 WT) or sumoylation-deficient BMAL1 (BMAL1 K259R) in HT22 cells. Sumoylation of BMAL1 was defected in a BMAL1 K259R mutant cDNA (Additional file 6: Figure S4A,B). We observed that Aβ-induced degradation of BMAL1 is mitigated by SUMO K259R mutant transfected HT22 cells (Figure 4G,H). Collectively, these data demonstrate that Aβ-induced BMAL1 degradation is due to the sumoylation of BMAL1.

### Aβ induces N-Cadherin CTF2-mediated degradation of CBP through the cleavage of N-Cadherin by γ-secretase

**Figure 5:**
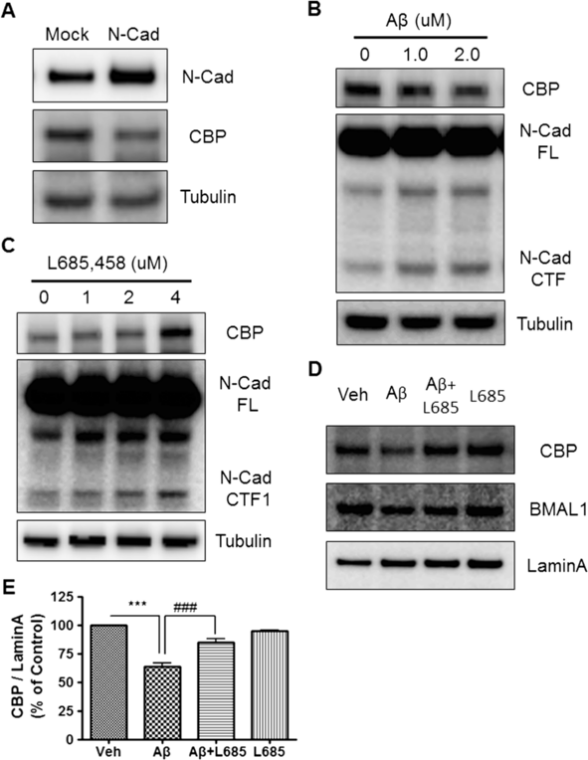
**The N-Cadherin mediated degradation of CBP was induced by Aβ. (A)** CBP degradation was mediated by N-Cadherin. HT22 cells transiently transfected with the N-Cadherin construct. CBP expression was significantly decreased by N-Cadherin transfection. **(B)** N-Cadherin CTF expression was increased by Aβ. HT22 cells were treated with Aβ (0 or 2 μM) for 24 h. Western blot analysis revealed that N-Cadherin CTF expression was elevated by Aβ in a dose dependent manner. **(C)** γ-secretase inhibitor treatment increased CBP expression. HT22 cells were treated with L-685,458. Western blot analysis showed that CBP expression was increased by L-685,458 in a dose dependent manner. **(D)** γ-secretase inhibitor treatment rescued Aβ-induced CBP degradation. HT22 cells were incubated with Aβ only, or Aβ combined with L-685,458, for 24 h. Western blot analysis revealed that Aβ-induced CBP degradation is ameliorated by L-685,458. **(E)** Densitometric analysis of CBP protein from three independent experiments. CBP was normalized to Lamin A. ^**^ *P* < 0.01, ^##^ *P* < 0.01.

It has been shown that the BMAL1-CLOCK-induced circadian rhythm is stimulated by CBP recruitment to the E-box of the *Per1* promoter [19]. Another study has shown that the N-Cadherin C-terminal fragment (CTF) binds to CBP and induces its degradation [25]. In current studies, N-Cadherin overexpression markedly reduced the steady state levels of CBP (Figure 5A). Next, we determined whether Aβ would affect the generation of the N-Cadherin CTF. We found that Aβ treatment resulted in increased levels of N-Cadherin CTF, resulting in decreased levels of CBP (Figure 5B). It has been shown that N-Cadherin is cleaved by ADAM10 to generate the N-Cadherin CTF1, which, in turn, is cleaved by γ-secretase to generate the N-Cadherin CTF2 [25]. To confirm the role of γ-secretase in the cleavage of N-Cadherin, we treated HT22 cells with the γ-secretase inhibitor L-685,458. We found that γ-secretase-mediated cleavage of CTF1 was significantly inhibited by L-685,458 treatment, resulting in the accumulation of CTF1 (Figure 5C). The reduced cleavage of CTF1 to CTF2 in L685,458-treated cells resulted in a dose-dependent increase of CBP levels, further suggesting that CTF2 induces CBP degradation. Since there have been several reports that Aβ induces γ-secretase activation in various systems, the current results suggest that treatment with a γ-secretase inhibitor might reduce Aβ-induced CBP degradation in HT22 cells, resulting in increased levels of CBP. Western blot analysis confirmed that Aβ-induced CBP degradation was significantly inhibited by L-685,458 treatment (Figure 5D,E). These results demonstrate that the cleavage of N-Cadherin by γ-secretase is the molecular mechanism underlying Aβ-induced degradation of CBP.

### Aβ modulates the expression of Per2 mRNA through Aβ-mediated degradation of BMAL1 and CBP

**Figure 6:**
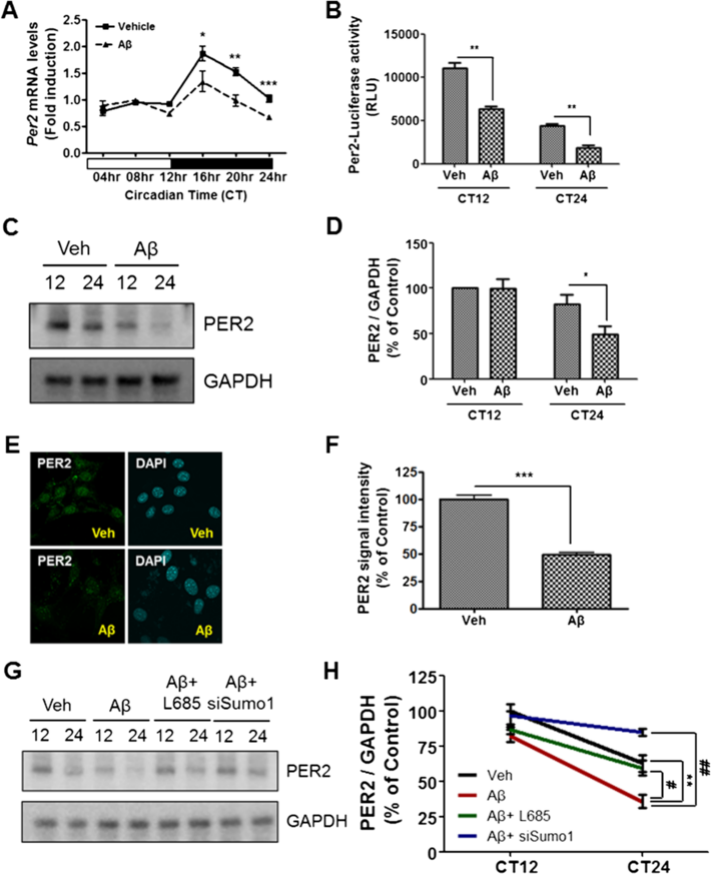
**Aβ modulated Per2 expression. (A)** Temporal expression of *Per2* mRNA in Aβ-treated HT22 cell. *Per2* mRNA levels in Aβ-treated HT22 cells were decreased and disrupted by circadian oscillation compared to vehicle-treated HT22 cells. Total RNA of HT22 cells was prepared at 4 h intervals. The mRNA level of *Per2* was quantified by real-time PCR. Data were represented as mean ± SEM. ^*^ *P* < 0.05, ^**^ *P* < 0.01, ^***^ *P* < 0.001. **(B)** HT22 cells were transiently transfected with *Per2*-luciferacse promoter construct. HT22 cells were treated with vehicle or Aβ (2 μM) for CT12, 24 after cell synchronization. *Per2*-promoter activity was significantly decreased by Aβ treatment. Data are represented as mean ± SEM. ^**^ *P* < 0.01. **(C)** HT22 cells were incubated with vehicle or Aβ (2 μM) for CT12, 24 after cell synchronization. Western blot analysis revealed that PER2 protein expression was decreased by Aβ treatment at CT24. **(D)** Densitometric analysis of PER protein from three independent experiments. PER2 was normalized to GAPDH. Data were represented as mean ± SEM. ^*^ *P* < 0.05. **(E)** Photomicrograph of immunostaining with PER2 in HT22 cells shows that PER2 immunofluorescence was significantly decreased by 2 μM of Aβ for 24 h. **(F)** Densitometric analysis of PER2 immunofluorescence from three independent experiments. Data were represented as mean ± SEM. ^**^ *P* < 0.01. **(G)** HT22 cells were treated Aβ with both L685,458 and siSumo1 for CT12, CT24 after cell synchronization. PER2 expression was significantly increased by L685,458 and siSumo1 treatment. **(H)** Densitometric analysis of PER protein from three independent experiments. PER2 was normalized to GAPDH. Data are represented as mean ± SEM. ^**^ *P* < 0.01, ^##^ *P* < 0.01, ^#^ *P* < 0.05.

It has been shown that BMAL1 and CLOCK induce the expressions of the *Per* and cryptochrome (*Cry*) genes, which, in turn, inhibit the interaction between BMAL1 and CLOCK [26,27]. Having shown that Aβ induced BMAL1 and CBP degradation, we next reasoned that Aβ may change the expression patterns of *Per* or *Cry*. To test this possibility, HT22 cells were treated with 2 μM Aβ after their cellular clocks were synchronized by pre-treatment with 0.2 μM Dex for 2 h. Total RNA from HT22 cells was isolated at 4 h intervals to analyze the temporal change in the level of *Per2* mRNA. The result showed that the oscillations in *Per2* mRNA levels were disrupted in Aβ-treated cells (Figure 6A). To examine whether Aβ treatment caused reduced binding of transcription factors to the *Per2* promoter, thereby disrupting PER2 protein expression and the oscillations of *Per2* mRNA level, we performed a *Per2-*promoter activity assay. HT22 cells were transiently transfected with the *Per2* promoter-luciferase cassette expressing luciferase from the *Per2* promoter. Transfected cells were synchronized, and subsequently incubated with vehicle or 2 μM Aβ for CT12 and 24. We found that the activity of the *Per2* promoter was significantly reduced in Aβ-treated cells at CT24 (Figure 6B). In addition, time-course immunoblotting analysis of synchronized cells revealed that the expression of PER2 protein was significantly down-regulated by Aβ treatment at CT24 (Figure 6C,D). Consistent with these results, immunofluorescence analysis showed that PER2 immunofluorescence in the nucleus of HT22 cells was significantly decreased by Aβ treatment (Figure 6E,F). To block sumoylation, siSumo1 was transfected. γ-secretase inhibitor, L685,458, was treated to block N-cadherin cleavage. In both cases, synchronized HT22 ells showed less oscillation of PER2 protein expression in CT12 and CT24 time points, which support the importance of both pathways in oscillation of clock gene (Figure 6G,H). These results indicate that Aβ modulates the expression of *Per2* mRNA through Aβ-mediated degradation of BMAL1 and CBP.

## Discussion

Although various studies have shown that circadian rhythms are disrupted in AD patients and animal models of AD [12,16,17,28], little is known about the mechanisms by which Aβ perturbs circadian rhythm. In the present study, we investigated the physiological functions and molecular mechanisms underlying the regulation of the circadian rhythm by Aβ. Our results show that 5XFAD mice, which overexpress Aβ and have amyloid deposits in their brains, have impaired circadian behavior associated with the altered expression of circadian clock molecules (Figures 1 and 2). To link a phenotypic impairment in circadian rhythm to the molecular mechanism of circadian clock genes, we further characterized the role of Aβ in transcriptional regulation, protein expression, and post-translational degradation of molecular clocks, such as BMAL1, CBP, and PER2. Our results suggest that Aβ enhances the degradation of BMAL1 and CBP (Figure 3), leading to the perturbed expression of PER2 at both the mRNA and protein level (Figure 6). Aβ-induced degradation of BMAL1 correlates with the interaction of SUMO1 with BMAL1 and Aβ-induced sumoylation of BMAL1 (Figure 4C,D). Aβ-induced cleavage of the N-Cadherin CTF promotes CBP degradation (Figure 5A,B), which is counteracted by a γ-secretase inhibitor (Figure 5D,E). These results collectively suggest that Aβ may be an important causative factor underlying the impaired circadian rhythm often observed in AD. Circadian rhythm is driven by an endogenous circadian clock, which oscillates with a period of approximately 24 h, and is entrainable to external cues. This fundamental biological property has been observed in a variety of organisms, such as mammals and plants, as well as fungi and cyanobacteria [29-31]. Circadian rhythm controls metabolism, hormone secretion, sleep, and cardiac function [32-34]. It is therefore not surprising that the disruption of the circadian rhythm is closely related to many diseases such as diabetes, obesity, bipolar disorder, cancer, and neurodegenerative diseases [6,22,32,35,36]. Recent studies have shown that circadian rhythm is perturbed in AD patients and mouse models of AD [1,7,9,11,33].

It is well established that core clock molecules regulate the circadian behavioral rhythm [37-39]. Since the suprachiasmatic nucleus (SCN), the central master clock, synchronizes central and peripheral oscillators to evoke circadian behavioral regulation, we explored whether the behavioral alterations in 5XFAD mice are mediated by alterations in expression of core clock molecules in the SCN. We found that the oscillation of circadian core clock molecules was disrupted in SCN tissue from two-month-old 5XFAD mice (Figures 1 and 2). To confirm Aβ levels in SCN, we measured Aβ levels in the SCN region of 2 month and 4 month old 5XFAD mice using Aβ ELISA (Additional file 7: Figure S5A). The Aβ level in SCN was lower than both Subiculum and Frontal cortex. Also, the Aβ level in SCN was increased in 4 month compared to 2 month old 5XFAD mice (Additional file 7: Figure S5B), suggesting that Aβ level in SCN is associated with circadian rhythm disruption. We also observed that in 8-month-old 5XFAD mice, the number of NeuN-positive cells was not altered in the SCN region (Additional file 8: Figure S6). Therefore, circadian behavior disruption in 5XFAD mice was not affected by SCN neuronal cell loss. Previous reports have indicated that circadian behavioral alterations in AD patients occur before the development of severe AD pathology. Based on these studies, we explored whether low levels of Aβ could affect oscillations in circadian molecules. We tested Aβ toxicity using MTT, Calcein-AM cell viability assays (Additional file 9: Figure S7), and found that 5 μM of Aβ treatment for 24 h significantly increased cell toxicity, whereas 2 μM of Aβ treatment, which is the concentration of Aβ we used in this study, did not induce cellular toxicity. Interestingly, 2 μM of Aβ regulated the stability of BMAL1 and CBP proteins (Figure 3). Protein sumoylation is a post-translational modification implied in diverse cellular processes, such as apoptosis, protein stability, transcriptional regulation, and nuclear-cytosolic transport. It is well known that sumoylation of BMAL leads to protein destabilization [23]. We found that the sumoylation of BMAL1 reinforced Aβ-dependent destabilization (Figure 4A and B), whereas knockdown of *Sumo1* did not induce Aβ-mediated BMAL1 degradation (Figure 4E and F). Sumoylation is known as an important post-translational modification during oxidative stress. The efficiency of sumoylation is affected by the exposure to stress condition, including heat shock, osmotic, and oxidative stress that enhance sumoylation [40]. Several lines of evidence showed that altered expression of SUMO enzymes and SNPs (single nucleotide polymorphism) of SUMO-related genes in AD patients’ brains [41]. Our previous study showed that Aβ induced the sumoylation of oxidative stress sensitive transcription factor, Sp1, to increase the binding of Sp1 to target gene [42]. Although the exact mechanism how Aβ induced BMAL1 sumoylation is not known at this moment, it is possible that Aβ activates the SUMO conjugating enzyme to add SUMO to BMAL1 through oxidative stress sensitive signaling pathway.

**Figure 7:**
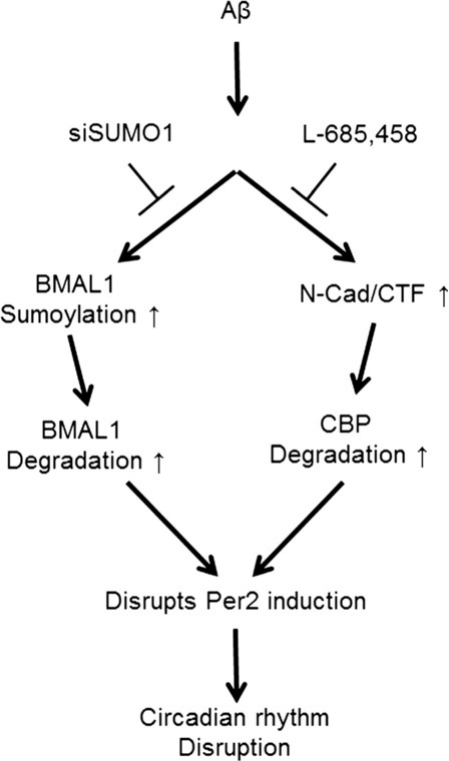
**Schematic diagram of the role of Aβ in circadian rhythm disruption.** This diagram describes the signaling cascade between AD and circadian rhythm, focusing on the role of Aβ. Based on our experimental data, Aβ can regulate BMAL1 and CBP degradation under conditions of Aβ-induced damage. BMAL1 degradation is mediated by Aβ-induced BMAL1 sumoylation. CBP degradation is mediated by Aβ-induced N-Cadherin cleavage. Thus, Aβ-induced BMAL1 and CBP expression results in disrupted PER2 oscillation and circadian rhythm disruption.

N-Cadherin (N-Cad) forms complexes with PS-1 [43], a calcium dependent cell adhesion glycoprotein containing five extracellular cadherin repeats with a highly conserved cytoplasmic tail and a transmembrane region. Both proteins are expressed in both neurons and synapses [43,44]. N-Cad is processed by γ-secretase-dependent cleavage to generate intracellular domain (ICD) peptide N-Cadherin CTF2 (N-Cad_CTF2) that forms complexes with CBP and promotes its proteasomal degradation, thus acting as a dominant repressor of CBP-mediated transcription [25]. CBP is a well-established key molecule for circadian clock gene expression, but little is known about its regulation despite a recent study by Ramendra et al. that has shown CBP degradation by Aβ treatment; however, since this CBP loss was not revealed at the level of *Cbp* gene expression, we confirmed that *Cbp* gene expression was not affected by Aβ treatment (Additional file 5: Figure S3B). Therefore, we hypothesized that Aβ-induced CBP degradation is mediated by N-Cad CTF. As expected, Aβ-mediated CBP degradation was prevented by the use of a γ-secretase inhibitor (Figure 5D,E). Figure 7 shows a schematic diagram, describing the role of Aβ in circadian rhythm disruption. BMAL1 and CBP function as upstream core clock molecules that regulate PER2 induction, leading to Aβ-induced circadian rhythm disruption.

Many papers have proposed that regulators of circadian rhythm could be therapeutic targets in neurodegenerative disorders [45,46]. In Huntington’s disease (HD), disturbed sleep is an important symptom. HD animal models, such as the R6/2 mouse, have also shown sleep disruption [47]. Alprazolam is used as a hypnotic drug to attempt to normalize sleep-wake cycles. Extended treatment with alprazolam has significantly improved cognitive function and clock gene oscillations in R6/2 mice. A recent study by Kang et al. revealed that circadian rhythm or sleep disturbance was significantly increased, and an orexin receptor antagonist markedly decreased Aβ plaque formation, in a mouse model of AD. Thus, disruptions to the sleep-wake cycle could exacerbate the progression of neurodegenerative disorders, and restoring a normal sleep cycle could have therapeutic potential in neurodegenerative diseases. Moreover, a recent study by Xie et al. showed that sleep drives metabolite clearance of waste molecules such as Aβ from the brain. They showed that sleep is associated with an increase in the volume of the interstitial space, resulting in a remarkable increase in convective exchange of interstitial fluid (ISF) with cerebrospinal fluid (CSF), which increased the rate of Aβ clearance during sleep [48].

## Conclusions

Our data collectively suggest that Aβ may play a critical functional role in circadian rhythm disruption through degradation of BMAL1 and CBP. Furthermore, this degradation was mediated by Aβ-induced sumoylation of BMAL1 and cleavage of N-Cadherin. Therefore, the prevention of Aβ-induced BMAL1 and CBP degradation, or improved Aβ clearance by restoration of the sleep-wake cycle, could be novel therapeutic targets for AD pathogenesis, and warrant further investigation.

## Methods

### AD transgenic mice

5XFAD mice express five familial AD mutations (three in human *APP*: K670N/M671L (Swedish) + I716V (Florida) + V717I (London) and two in human *PSEN1*: M146L + L286V), with neuronal expression driven by the *Thy-1* promoter [49]. These animals were purchased from Jackson Laboratory (Bar Harbor, ME; catalog number 006554). Treatment and maintenance of the animals were conducted in accordance with the Guide for the Care and Use of Laboratory Animals (NIH publication No. 85–23, revised 1985) and the Animal Care and Use Guidelines of Seoul National University, Seoul, Korea.

### Monitoring home cage activity and body temperature

The monitoring of mouse home cage activity and body temperature was performed as previously described [50]. A G2 E-mitter probe (Mini Mitter, Bend, OR) was surgically inserted under the skin on the dorsum of the neck [51], and home cage activity and body temperature were continuously monitored using the Activity Monitoring System (Mini Mitter, Bend, OR). Data were reported at 6 min intervals using the VitalViewH Data Acquisition System. Actograms from individually monitored mice were obtained using ActiViewH software. To generate the daily actogram pattern for each mouse, monitoring results displayed as an MS Excel file for the whole time period were either averaged (in case of body temperature), or summed (in case of home cage activity) as 1 h bins, and the resulting 28 day profiles were pooled according to the indicated circadian time (CT).

### Cell culture and transfection

HT22 mouse hippocampal cells and Cos7 monkey kidney cells were maintained in Dulbecco’s Modified Eagle’s Medium (DMEM; HyClone Laboratories, Salt Lake City, UT) supplemented with 10% fetal bovine serum (FBS; HyClone Laboratories, Salt Lake City, UT), 100 U/mL penicillin, and 0.1 mg/mL streptomycin (Sigma-Aldrich, St Louis, MO) at 37°C in a 5% CO_2_ incubator. The control siRNA (SC-37007) and siRNA against SUMO1 (SC-36574) were purchased from Santa Cruz Biotechnology (Santa Cruz, CA). cDNA expression vectors for pcDNA™ 3.1-mouse CBP, Per2-Luc, Myc-BMAL1, VC-BMAL1 and VN-CBP constructs were generously gifted from Dr. Kyungjin Kim (Seoul National University, Korea). Transient transfections were performed using Lipofectamine™ LTX (Invitrogen, Carlsbad, CA) for cDNA and RNAimax for siRNA (Invitrogen, Carlsbad, CA) according to the instructions provided by the manufacturer.

### Reagents and antibodies

Aβ, Dexamethasone (Dex), MG-132, Cycloheximide (CHX), and L-685,458 were purchased from the American Peptide Company (Sunnyvale, CA), Sigma-Aldrich (St. Louis, MO), and Calbiochem (La Jolla, CA). The following antibodies were used for immunodetection: anti-BMAL1, anti-CBP (Santa Cruz Biotechnology Inc., Santa Cruz, CA), anti-PER2 (Alpha Diagnostic International Inc., San Antonio, TX), anti-Sumo1 (Cell Signaling, Beverly, MA), anti-N-Cadherin (BD Biosciences, San Jose, CA), anti-Lamin A (Abcam, Cambridge, MA), and anti-α-Tubulin (Sigma-Aldrich, St Louis, MO).

### Western blot analysis

Vehicle or Aβ treated cells were washed twice with ice-cold PBS, scraped and resuspended in RIPA buffer (50 mM Tris–HCl, pH 7.4; 150 mM NaCl; 1% Nonidet P-40; 0.1% SDS; 0.5% deoxycholic acid sodium salt) containing a protease inhibitor cocktail (Sigma-Aldrich, St Louis, MO) and incubated on ice for 15 min. After centrifugation at 17,000 g for 15 min, the protein concentration was determined using a BCA assay kit (Pierce, Rockford, IL). Equal amounts of protein were resolved using Tris-glycine polyacrylamide gels. Proteins were transferred to PVDF membranes (Millipore, Billerica, MA), and incubated with antibodies against target proteins. The bands were visualized using enhanced chemiluminescence (ECL; Amersham Pharmacia Biotech, Buckinghamshire, England) with an image analyzer (LAS-3000; Fuji, Tokyo, Japan).

### Immunocytochemistry

Immunofluorescence using the primary antibodies for BMAL1, CBP and PER2 was performed as previously described [52]. Briefly, vehicle- or Aβ-treated cells were washed twice with ice-cold PBS. Following fixation (4% paraformaldehyde for 15 min), cells were washed with PBS, permeabilized (0.5% Triton X-100), blocked for 1 h (PBS containing 2% horse serum, 2% goat serum, and 2% fetal bovine serum) and incubated with primary antibodies overnight at 4°C, followed by incubation with Alexa Fluor® 488- or Alexa Fluor® 594-conjugated secondary antibodies (1:500, Invitrogen, Carlsbad, CA) for 1 h. After washing, cells were counterstained for 10 min with 4′-6-Diamidino-2-phenylindole (DAPI, 1:5,000; Sigma-Aldrich, St Louis, MO). Fluorescent signals were visualized using confocal microscopy (FV10vi, Olympus, Tokyo, Japan).

### Luciferase reporter gene assay

For luciferase reporter gene assays, the Per2-Luc construct was transfected using Lipofectamine™ LTX and Plus reagent (Invitrogen, Carlsbad, CA) according to the manufacturer’s protocol. Approximately 24 h after transfection, cells were lysed in Passive Lysis Buffer (PLB; Promega, Madison, WI), and luciferase activity in the cell extracts was measured using the Dual-Luciferase Reporter Assay System (Promega, Madison, WI).

### Plasmids and BiFC assay

Bimolecular fluorescence complementation (BiFC) assays were the same as previously reported [19]. Cos7 cells were transiently transfected with VC-BMAL1 and VN-CBP. At 12 h post-transfection with VN–CBP and VC–BMAL1, cells were serum starved for 12 h, and incubated with either vehicle or Aβ for 24 h following cell synchronization. For BiFC quantitative analyses, the cells showing nuclear signals were measured among BiFC signal-positive cells after serum shock synchronization in the vehicle- or Aβ-treated cells.

### Immunoprecipitation

Immunoprecipitation (IP) was performed as previously described [53]. Vehicle or Aβ-treated HT22 cells were lysed in RIPA buffer (50 mM Tris–HCl, pH 7.5; 150 mM sodium chloride; 0.5% sodium deoxycholate; 0.1% SDS; 1% Triton X-100; 2 mM EDTA) containing protease inhibitors (Sigma-Aldrich). Lysates were centrifuged at 17,000 g for 15 min. Equal amounts of protein were precipitated with the anti-BMAL1 antibody at 4°C overnight on a rotator. Protein A/G agarose beads (Santa Cruz Biotechnology) were added to each sample and incubated at 4°C for 1 h. IPs were collected by centrifugation and then washed four times with the same buffer. The agarose beads were resuspended in 10 μL of 5× sample buffer and incubated at 95°C for 10 min to release the proteins. After a pulse spin, the supernatants were loaded on a Tris-glycine polyacrylamide gel for western blotting.

### Real-time PCR

RNA was isolated using the RNeasyR Plus Mini Kit (Qiagen, Valencia, CA) and cDNA was generated using the RevertAid First Strand cDNA Synthesis Kit (Fermentas, Glen Burnie, MD). Real-time PCR was performed on the cDNA samples using ABI StepOne 2.1 (Applied Biosystems, Foster City, CA). The primers used were described in Kohsaka et al. [32]. The following primers were used: mouse *Bmal1*, forward, 5′-CCACCTCAGAGC CATTGATACA-3′; and reverse, 5′-GAGCAGGTTTAGTTCCACTTTGTCT-3′; mouse *Per2*, forward, 5′-TGTGCGATGATGATTCGTGA-3′; and reverse, 5′-GGTGAAGGTACGTTT GGTTTGC-3′; mouse *Cbp*, forward, 5′-CACAGAACCAGTTTCCATCATCCAGT-3′; and reverse, 5′-CATGTTCAGAGGGTTAGGGAGAGCA-3′; mouse *Gapdh*, forward, 5′-ACAG CCGCATCTTCTTGTGCAGTG-3′; and reverse, 5′-GGCCTTGACTGTGCCGTTGAATTT-3′. The *Gapdh* gene was used as an endogenous control to standardize the amount of RNA in each reaction.

### Preparation of Aβ peptide

Aβ peptide (American peptide, Sunnyvale, CA, USA) was prepared as previously described [54]. Aβ peptide was dissolved in 1,1,1,3,3,3,-hexafluoro-2-propanol (HFIP) (Sigma-Aldrich). HFIP solution was divided and evaporated by vacuum (SpeedVac Concentrator; Savant Instruments, Hyderabad, India). Aβ peptide was maintained at deep freezer and dissolved with anhydrous dimethyl sulfoxide (Sigma-Aldrich).

### Aβ42 ELISA

ELISA was performed as previously described [55]. ELISA was performed for quantification of Aβ42 in the two-month-old 5XFAD mice. Brain tissue lysed in RIPA buffer using sonicatior and ultracentrifuged at 100,000 g at 4°C for 1 hr. The pellet was resuspended in 70% formic acid solution and ultracentrifuged at 100,000 g at 4°C for 1 hr. Protein concentrations were measured using a BCA kit (Thermo scientific). ELISA samples were run in duplicate on Aβ42 ELISA following the protocol of the manufacturer (IBL, Japan). OD at 450 nm were read on a plate reader (Powerwave XS; BIO-TEK, Winooski, VT, USA).

### Statistical analysis

For statistical analysis, a Student *t*-test or one-way ANOVA was performed, followed by Tukey’s *post-hoc* test. (^*^P < 0.05, ^**^P < 0.01, ^***^P < 0.001 or ^###^*P* < 0.001) using GraphPad Prism Version 4.0 (GraphPad Software, San Diego, CA). The factor analysis between groups was using two-way ANOVA with Bonferroni posttests (^*^P < 0.05, ^**^P < 0.01, ^***^P < 0.001 or ^****^P < 0.001).

## Additional files

Additional file 1: Figure S1. Disrupted circadian rhythm in 5XFAD mice. (A-D) Representative actograms of body temperature and home cage activity of littermate and 5XFAD mice. (A & B) Two-month-old 5XFAD mice exhibited distinct peaks in home cage activity and body temperature at LD and DD cycle. (C & D) Eight-month-old 5XFAD mice exhibited distinct peaks in home cage activity and body temperature at LD and DD cycle. Additional file 2: Table S1. Summary of HCA rythms. Additional file 3: Table S2. Summary of BT rythms. Additional file 4: Figure S2. The disruption of BMAL1 and CBP interaction was mediated by Aβ. (A) The interaction of CBP with BMAL1 was decreased by Aβ in HT22 cells. HT22 cells were transfected with CBP and BMAL1 cDNA constructs. Twenty-four hours after transfection, cells were incubated with vehicle or Aβ for 24 h. The cells were harvested and lysed in lysis buffer, followed by immunoprecipitation with the anti-BMAL1 antibody. The immunoprecipitated product was analyzed with anti-CBP antibody using western blotting. Additional file 5: Figure S3. Temporal expression of *Bmal1* and *CBP* mRNA levels in Aβ-treated HT22 cells. (A & B) *Bmal1 and CBP* mRNA levels in Aβ-treated HT22 cells did not show circadian oscillations compared with vehicle-treated HT22 cells. The total RNA of HT22 was prepared at 4 h intervals. The mRNA level of *Bmal1 and CBP* was quantified by real-time PCR. Additional file 6: Figure S4. Disrupted sumoylation of BMAL1 in the BMAL1 K259R mutant overexpressing cells. (A & B) HT22 cells were transiently transfected with BMAL1 WT or K259R cDNA construct. Twenty-four hours after transfection, cells were harvested and lysed with lysis buffer, followed by immunoprecipitation with anti-BMAL1 antibody. The Immunoprecipitated product was analyzed with anti-SUMO1 antibody. Additional file 7: Figure S5. Measuring Aβ levels in the SCN of 5XFAD mice. (A) RIPA soluble Aβ levels of two-month-old 5XFAD mice. (B) RIPA soluble Aβ levels of four-month-old 5XFAD mice. Additional file 8: Figure S6. Neuronal cell density was not altered in the SCN region of both littermate and 5XFAD mice. (A) NeuN-positive neuronal cell staining in the SCN region of eight-month-old littermate mice. (B) NeuN-positive neuronal cell staining in the SCN region of eight-month-old 5XFAD mice. Additional file 9: Figure S7. Aβ-induced cellular toxicity was measured by MTT and Calcein-AM cell viability assays. (A & B) HT22 cells were incubated with Aβ at various doses and times. Using the MTT and Calcein-AM assay, treatment of 5 μM Aβ for 24 h induced cellular toxicity. Data are represented as mean ± SEM. ^*^ *P* < 0.05, ^**^ *P* < 0.01, ^***^ *P* < 0.001.

## Abbreviations

Aβ: Amyloid-beta
AD: Alzheimer’s disease
BiFC: Bimolecular fluorescence complementation
BMAL1: Aryl hydrocarbon receptor nuclear translocator-like
CBP: Creb-binding protein
CHX: Cycloheximide
CRY: Cryptochrome
CSF: Cerebrospinal fluid
Dex: Dexamethasone
ICD: Intracellular domain
ISF: Interstitial fluid
PER2: Period circadian protein homolog 2
SCN: Suprachiasmatic nucleus
