# Supplementary figures and images for: Aβ-induced degradation of BMAL1 and CBP leads to circadian rhythm disruption in Alzheimer’s disease
Source: Mol Neurodegener. 2015 Mar 19;10:13. doi: 10.1186/s13024-015-0007-x (PMC4404698; doi:10.1186/s13024-015-0007-x)

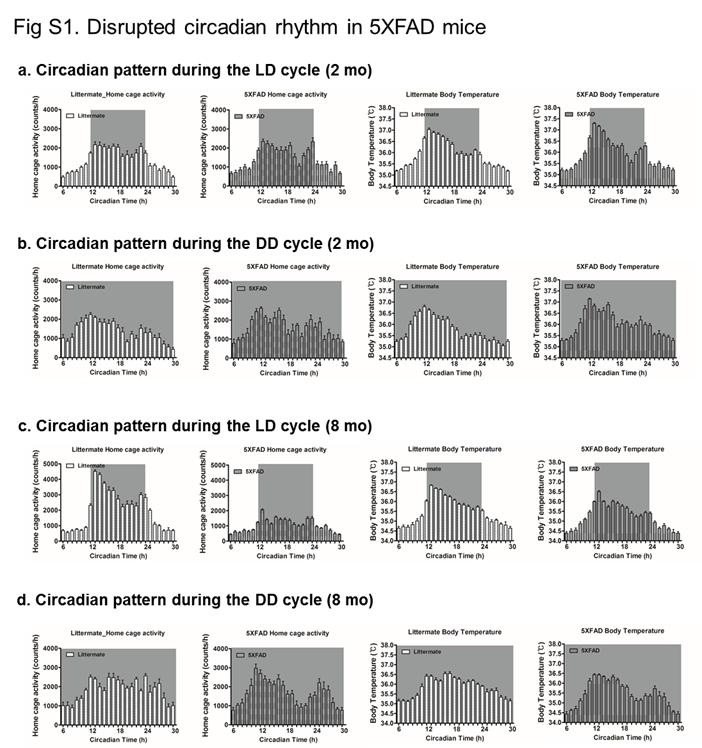

Supplement: Additional file 1: Figure S1. — Disrupted circadian rhythm in 5XFAD mice. (A-D) Representative actograms of body temperature and home cage activity of littermate and 5XFAD mice. (A & B) Two-month-old 5XFAD mice exhibited distinct peaks in home cage activity and body temperature at LD and DD cycle. (C & D) Eight-month-old 5XFAD mice exhibited distinct peaks in home cage activity and body temperature at LD and DD cycle. [file 13024_2015_7_MOESM1_ESM.tiff]

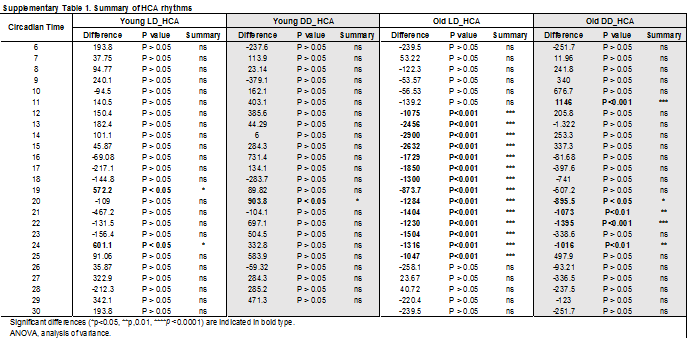

Supplement: Additional file 2: Table S1. — Summary of HCA rythms. [file 13024_2015_7_MOESM2_ESM.tiff]

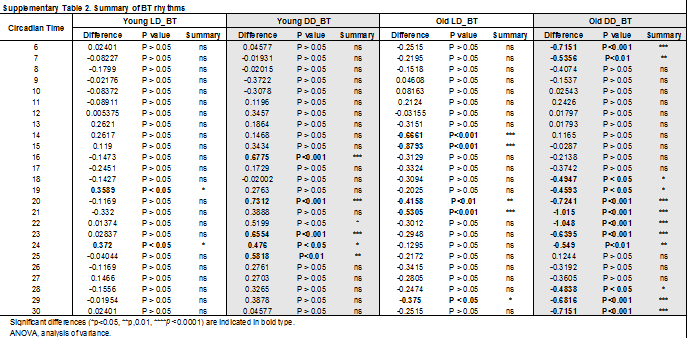

Supplement: Additional file 3: Table S2. — Summary of BT rythms. [file 13024_2015_7_MOESM3_ESM.tiff]

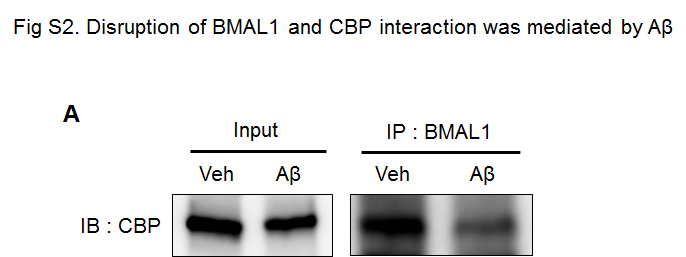

Supplement: Additional file 4: Figure S2. — The disruption of BMAL1 and CBP interaction was mediated by Aβ. (A) The interaction of CBP with BMAL1 was decreased by Aβ in HT22 cells. HT22 cells were transfected with CBP and BMAL1 cDNA constructs. Twenty-four hours after transfection, cells were incubated with vehicle or Aβ for 24 h. The cells were harvested and lysed in lysis buffer, followed by immunoprecipitation with the anti-BMAL1 antibody. The immunoprecipitated product was analyzed with anti-CBP antibody using western blotting. [file 13024_2015_7_MOESM4_ESM.tiff]

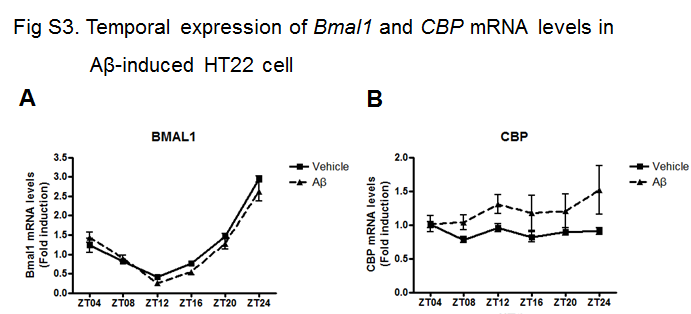

Supplement: Additional file 5: Figure S3. — Temporal expression of Bmal1 and CBP mRNA levels in Aβ-treated HT22 cells. (A & B) Bmal1 and CBP mRNA levels in Aβ-treated HT22 cells did not show circadian oscillations compared with vehicle-treated HT22 cells. The total RNA of HT22 was prepared at 4 h intervals. The mRNA level of Bmal1 and CBP was quantified by real-time PCR. [file 13024_2015_7_MOESM5_ESM.tiff]

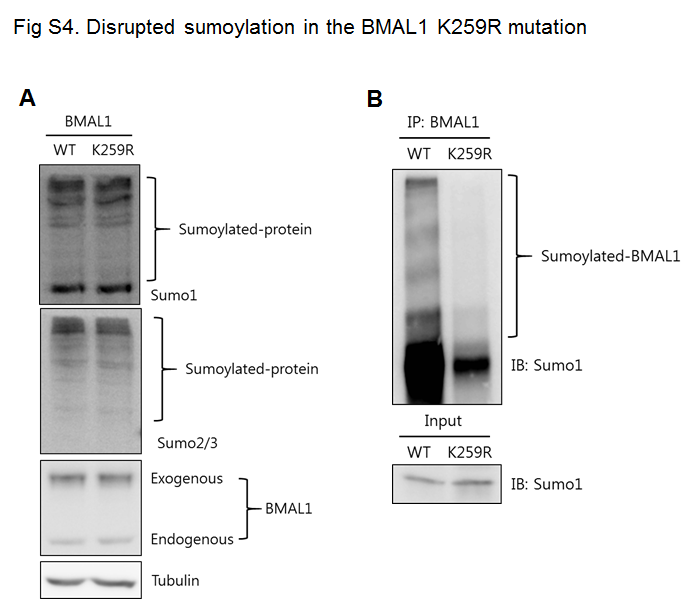

Supplement: Additional file 6: Figure S4. — Disrupted sumoylation of BMAL1 in the BMAL1 K259R mutant overexpressing cells. (A & B) HT22 cells were transiently transfected with BMAL1 WT or K259R cDNA construct. Twenty-four hours after transfection, cells were harvested and lysed with lysis buffer, followed by immunoprecipitation with anti-BMAL1 antibody. The Immunoprecipitated product was analyzed with anti-SUMO1 antibody. [file 13024_2015_7_MOESM6_ESM.tiff]

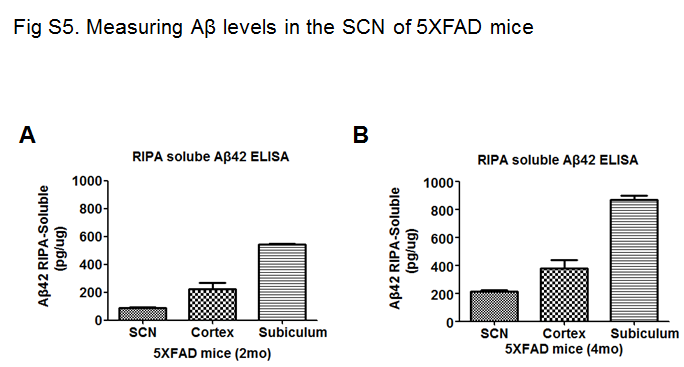

Supplement: Additional file 7: Figure S5. — Measuring Aβ levels in the SCN of 5XFAD mice. (A) RIPA soluble Aβ levels of two-month-old 5XFAD mice. (B) RIPA soluble Aβ levels of four-month-old 5XFAD mice. [file 13024_2015_7_MOESM7_ESM.tiff]

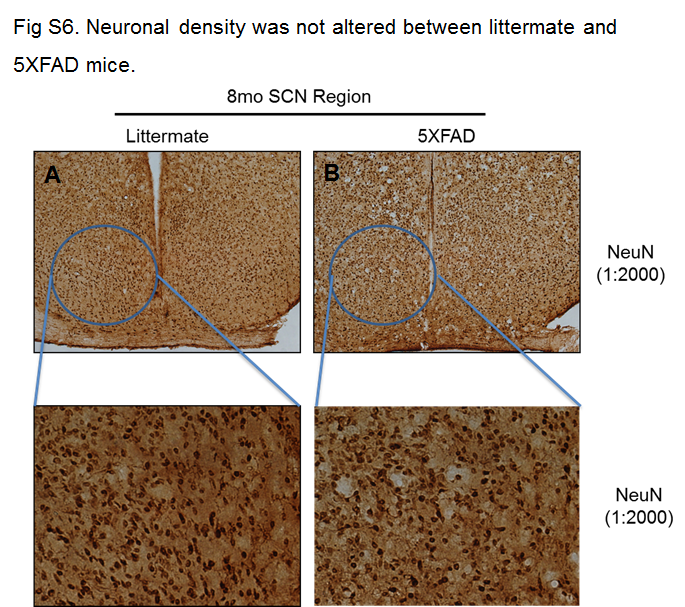

Supplement: Additional file 8: Figure S6. — Neuronal cell density was not altered in the SCN region of both littermate and 5XFAD mice. (A) NeuN-positive neuronal cell staining in the SCN region of eight-month-old littermate mice. (B) NeuN-positive neuronal cell staining in the SCN region of eight-month-old 5XFAD mice. [file 13024_2015_7_MOESM8_ESM.tiff]

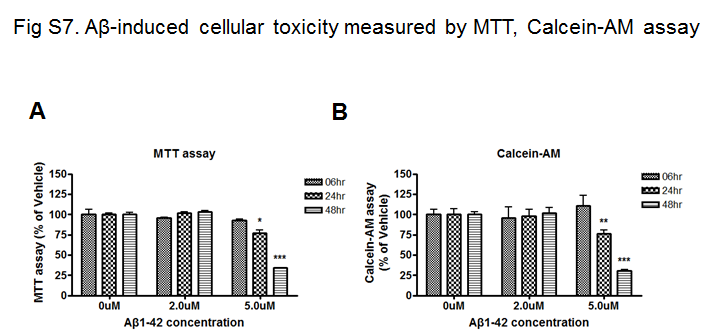

Supplement: Additional file 9: Figure S7. — Aβ-induced cellular toxicity was measured by MTT and Calcein-AM cell viability assays. (A & B) HT22 cells were incubated with Aβ at various doses and times. Using the MTT and Calcein-AM assay, treatment of 5 μM Aβ for 24 h induced cellular toxicity. Data are represented as mean ± SEM. * P < 0.05, ** P < 0.01, *** P < 0.001. [file 13024_2015_7_MOESM9_ESM.tiff]
